# Decellularized bone matrix-enriched 3D-printed GelMA scaffold as a cell-homing platform: analysis using an artificial pulp chamber model

**DOI:** 10.1007/s00784-026-06761-7

**Published:** 2026-02-09

**Authors:** Isabela Sanches Pompeo da Silva, Vitor de Toledo Stuani, Ester Alves Ferreira Bordini, Fernanda Balestrero Cassiano, Erika Soares Bronze-Uhle, Priscila Toninatto Alves de Toledo, Lídia de Oliveira Fernandes, Josimeri Hebling, Carlos Alberto de Souza Costa, Diana Gabriela Soares

**Affiliations:** 1 Department of Operative Dentistry, Endodontics, and Dental Materials, University of São Paulo – USP, Bauru School of Dentistry, Alameda Doutor Octávio Pinheiro Brisolla, 9-75, Bauru, SP Brazil; 2 Department of Prosthodontics and Dental Materials, University of São Paulo – USP, Ribeirão Preto School of Dentistry, Ribeirão Preto, Brazil; 3 Department of Pediatric Dentistry, School of Dentistry of Araraquara, São Paulo State University (Unesp), Street Humaitá, 1680 - Araraquara, São Paulo, 14801-385 Brazil; 4 Department of Physiology and Pathology, Araraquara School of Dentistry, São Paulo State University – UNESP, Araraquara, SP Brazil

**Keywords:** Tissue engineering, Hydrogels, Decellularized extracellular matrix, Dentin, Gelatin

## Abstract

**Objective:**

The aim of the study was to develop and evaluate bio-printed hydrogels based on gelatin methacrylate (GelMA) combined with different proportions of decellularized bovine bone matrix microparticles (BMdc).

**Methods:**

GelMA hydrogels were synthesized and incorporated with decellularized bovine bone matrix (BMdc) at 1% by weight. 3D scaffolds were fabricated through extrusion, with varying infill densities (40%, 50%, and 60%), followed by photoactivation. Biological analyses included cell viability (Live/Dead assay), and proliferation (Alamar Blue assay), as well as osteogenic differentiation (ALP activity and Alizarin Red staining) over a 21-day period in HDPCs. Porosity and pore size were assessed with Rhodamine B staining, and cell migration to scaffolds was evaluated in a biomimetic artificial pulp chamber model. Data were analyzed with one-way ANOVA and Tukey’s test (*p* < 0.05).

**Results:**

Scaffolds with the highest porosity and the largest pore size in comparison with other groups was detected in the 40% infill group (*p* < 0.05). Cells in the 50% and 60% infill groups exhibited higher viability, proliferation, and osteogenic differentiation, especially when BMdc particles were incorporated (*p* < 0.05). The greatest cell migration at the artificial pulp chamber model was observed in the 60% infill group in association with BMdc particles (*p* < 0.05).

**Conclusions:**

In summary, 3D-printed GelMA-BMdc hydrogel with 60% infill is a cytocompatible biomaterial capable of inducing cell adhesion, odontogenic differentiation, and mineralization. This innovative biomaterial shows potential for future direct pulp capping applications.

**Graphical Abstract:**

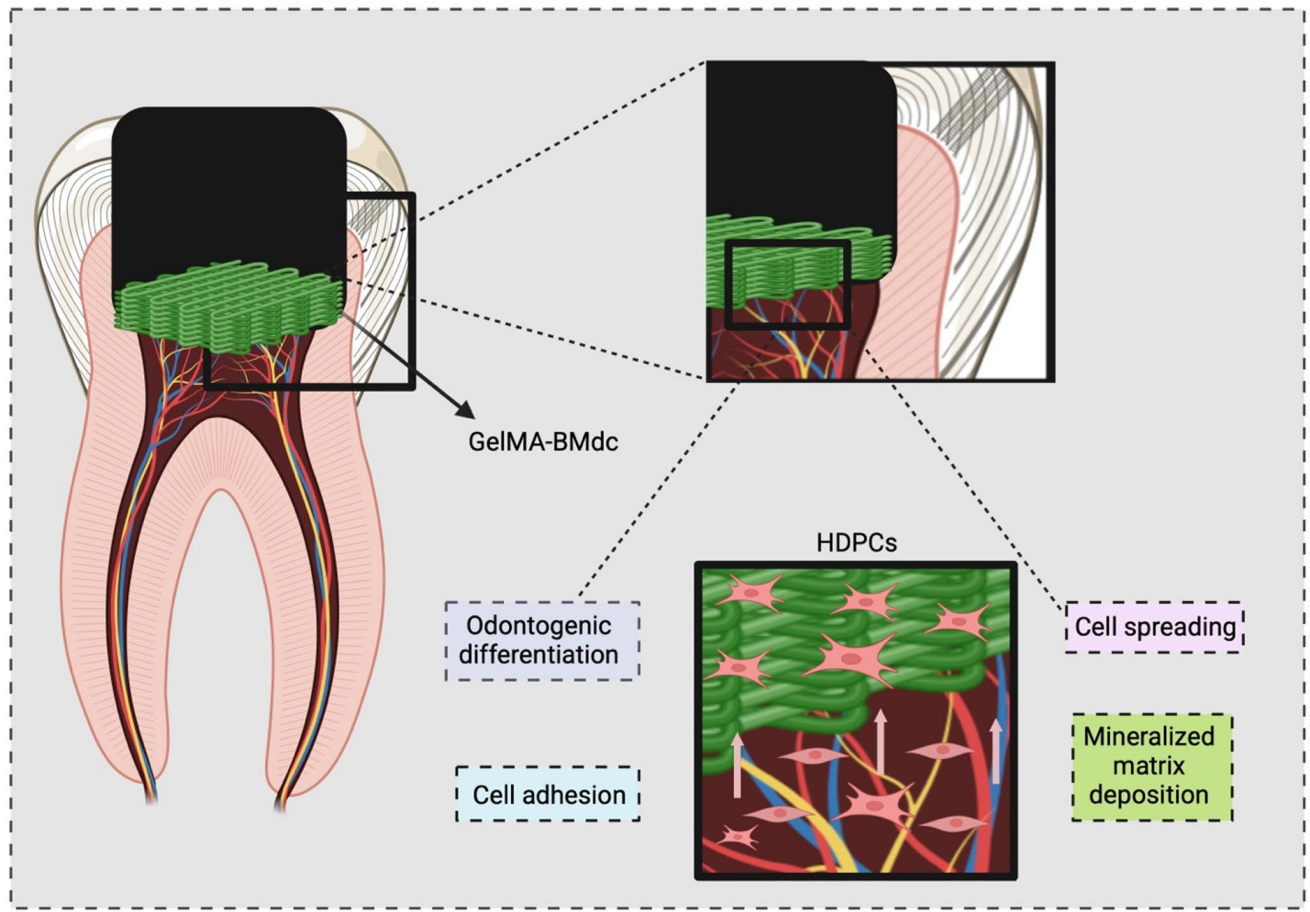

**Supplementary Information:**

The online version contains supplementary material available at 10.1007/s00784-026-06761-7.

## Introduction

The cell-homing strategy represents a promising approach for pulp-dentin regeneration, particularly in direct capping therapies. Biomaterials containing chemotactic signaling molecules can facilitate the migration and differentiation of pulp cells into odontoblast-like cells, thereby promoting dentin matrix synthesis and deposition [1–4]. Gelatin Methacryloyl (GelMA), a natural polymer derived from collagen, offers numerous advantages for tissue engineering applications due to its biocompatibility, low immunogenicity, and excellent biodegradability [2, 5–9]. Post-implantation, GelMA degrades enzymatically, enabling the controlled release of bioactive molecules and gradual replacement with newly formed tissue [10, 11]. 

A key feature of GelMA is its ability to form a stable hydrogel network through photoinitiator-induced crosslinking and light exposure, allowing precise control over the gelation process. This property of GelMA supports 3D printing techniques and enables the fabrication of complex structures [6, 12, 13]. Furthermore, GelMA incorporates cell-responsive gelatin-derived molecules, such as matrix metalloproteinases (MMPs) and RGD (Arg-Gly-Asp) sequences, which promote cell attachment, spreading, and proliferation within the hydrogel matrix [12, 14]. Due to its close similarity to the natural extracellular matrix (ECM), GelMA can be modified or functionalized with bioactive molecules, such as growth factors, peptides, and drugs, to enhance specific cellular responses [15–19]. 

Decellularized extracellular matrices (dECMs) have emerged as potent signaling factors in tissue regeneration. These matrices preserve essential proteins, such as collagen, fibronectin, and laminin, as well as growth factors that provide crucial signals for cell adhesion, migration, proliferation, and differentiation [20–22]. A study by Paduano et al. (2016) [23] demonstrated that a hydrogel scaffold derived from bone dECM significantly increased the expression of dentin sialophosphoprotein (DSPP), dentin matrix protein 1 (DMP-1), and extracellular matrix phosphoglycoprotein (MEPE), as well as mineral deposition in dental pulp stem cells (DPSCs) after 21 days of culture. In a current study from our group, Da Silva et al. (2024) [24] assessed the bioactivity of GelMA combined with decellularized extracellular bovine bone matrix (BMdc). The authors showed that such combination produces a porous and stable hydrogel that enhances odontoblast differentiation and mineral deposition in human dental pulp cells (HDPCs), demonstrating its potential for pulp-dentin regeneration.

The optimal pore size and porosity of scaffolds for mineralized tissue regeneration can be achieved through 3D printing, an innovative technique that allows for the modulation of hydrogel microstructures to create porous scaffolds. This method uses a computer-aided design (CAD) model to guide the 3D printer in depositing bioink layer by layer, enabling precise scaffold construction [25–27]. Recently, Yang et al. (2023) [28] combined porcine dental follicle-derived dECM with methacrylate gelatin (GelMA) to form a GelMA/dECM cell-laden 3D bioprinted scaffold, which exhibited excellent mechanical properties, printability, biocompatibility, and capacity for inducing fibrogenesis and osteogenic differentiation in vivo. The potential for pulp-dentin regeneration was further explored by Cunha et al. (2023),[29] using a 3D-printed GelMA microgel supplemented with dentin matrix molecules, which successfully induced odontoblastic differentiation and mineral deposition.

In this study, a bioactive hydrogel was developed based on GelMA, combined with BMdc, as a bioink for the fabrication of 3D-printed scaffolds, with the aim of creating a biomaterial suitable for direct pulp capping. To assess the potential of the 3D-printed hydrogel in a cell-homing strategy, an artificial pulp chamber (APC) biomimetic model was used.

## Materials and methods

### Gelatin methacryloyl (GelMA) hydrogel synthesis

Gelatin methacryloyl (GelMA) was prepared by dissolving type A porcine gelatin (Sigma-Aldrich, St. Louis, MO, USA) in phosphate-buffered saline (PBS; pH 7.4; Gibco, Invitrogen, Carlsbad, CA, USA) at a concentration of 10% (w/v) at 50 °C. Methacrylic anhydride was then added to the solution, and the mixture was stirred for 2 h. Following this, 100 mL of PBS (Gibco, Invitrogen) was added, and the resulting solution was dialyzed in deionized water for 5 days with two daily water changes. After dialysis, the final solution was filtered (0.22 μm), frozen at −80 °C for 2 days, and lyophilized (150 × 10⁻³ Mbar; FreeZone, Labconco Corporation, Kansas City, MO, USA) at −80 °C for 5 days. The lyophilized GelMA was then dissolved in PBS (Gibco, Invitrogen) at a concentration of 15% (w/v), and 0.075% (w/v) lithium phenyl-2,4,6-trimethylbenzoylphosphinate (LAP; Sigma-Aldrich) photoinitiator was added to formulate the hydrogel.

### Decellularized bovine bone matrix (BMdc) obtention

Samples of the trabecular area of clavicular bovine bone (Protocol no. 002/2023, Ethics Committee on the Use of Animals, Bauru School of Dentistry, Bauru, SP, Brazil) were subjected to a decellularization protocol described by Da Silva et al. (2024) [24]. The samples were incubated in the following sequence of reagents: (1) PBS (Gibco, Invitrogen) + 0.1% EDTA (Sigma-Aldrich) at 25 °C for 1 h; (2) PBS (Gibco, Invitrogen) + 10 mM Tris (Invitrogen, Thermo-Fisher Scientific, Eugene, OR, USA) + 0.1% EDTA (Sigma-Aldrich) at 4 °C overnight; (3) PBS (Gibco, Invitrogen) + 10 mM Tris (Invitrogen) + 0.5% sodium dodecyl sulfate (SDS; Sigma-Aldrich) for 12 h; (4) Seven successive washes in PBS (Gibco, Invitrogen); (5) Enzymatic solution containing 50 U/mL of DNase (Thermo-Fisher Scientific) + 1 U/mL of RNase (Thermo-Fisher Scientific) + 10 mM Tris (Invitrogen) for 3 h at 37 °C. Afterward, the samples were frozen at −20 °C and subjected to overnight lyophilization at −80 °C (150 × 10⁻³ Mbar; FreeZone, Labconco Corporation). Decellularization was verified by nuclei fluorescence staining, analysis of DNA content and total protein quantification (supplemental material). To achieve a particle size of 75 μm, the samples were macerated using a crucible and pestle, then sieved. To prepare the GelMA-BMdc hydrogel, the bone particles were added and mixed into the GelMA hydrogel at 1% by weight. This concentration was selected based on Silva et al. (2024), who demonstrated that at this concentration, GelMA-BMdc supported dental pulp cell viability and proliferation, exhibiting bioactive potential.

### Hidrogel preparation

A 15% (w/v) GelMA solution was prepared by dissolving lyophilized GelMA in PBS (Gibco, Invitrogen) at 50 °C, with 0.075% (w/v) LAP (Sigma-Aldrich) included. The mineral phase BMdc was incorporated into the GelMA solution at concentrations of 1% (w/v) under vortex for 1 min. Injected samples (non-printed controls) were prepared by injecting 100 µL of the hydrogel from each group into the wells of 96-well plates. This was followed by photo-polymerization for either 30–15 s, using an LED light with a wavelength range of 385 to 515 nm (1,200 mW/cm2; Bluephase N, Ivoclar-Vivadent, Buffalo, NY, USA). The final samples measured 6 mm in diameter and 2.5 mm in thickness.

### Three-dimensional printed scaffolds

**Fig. 1:**
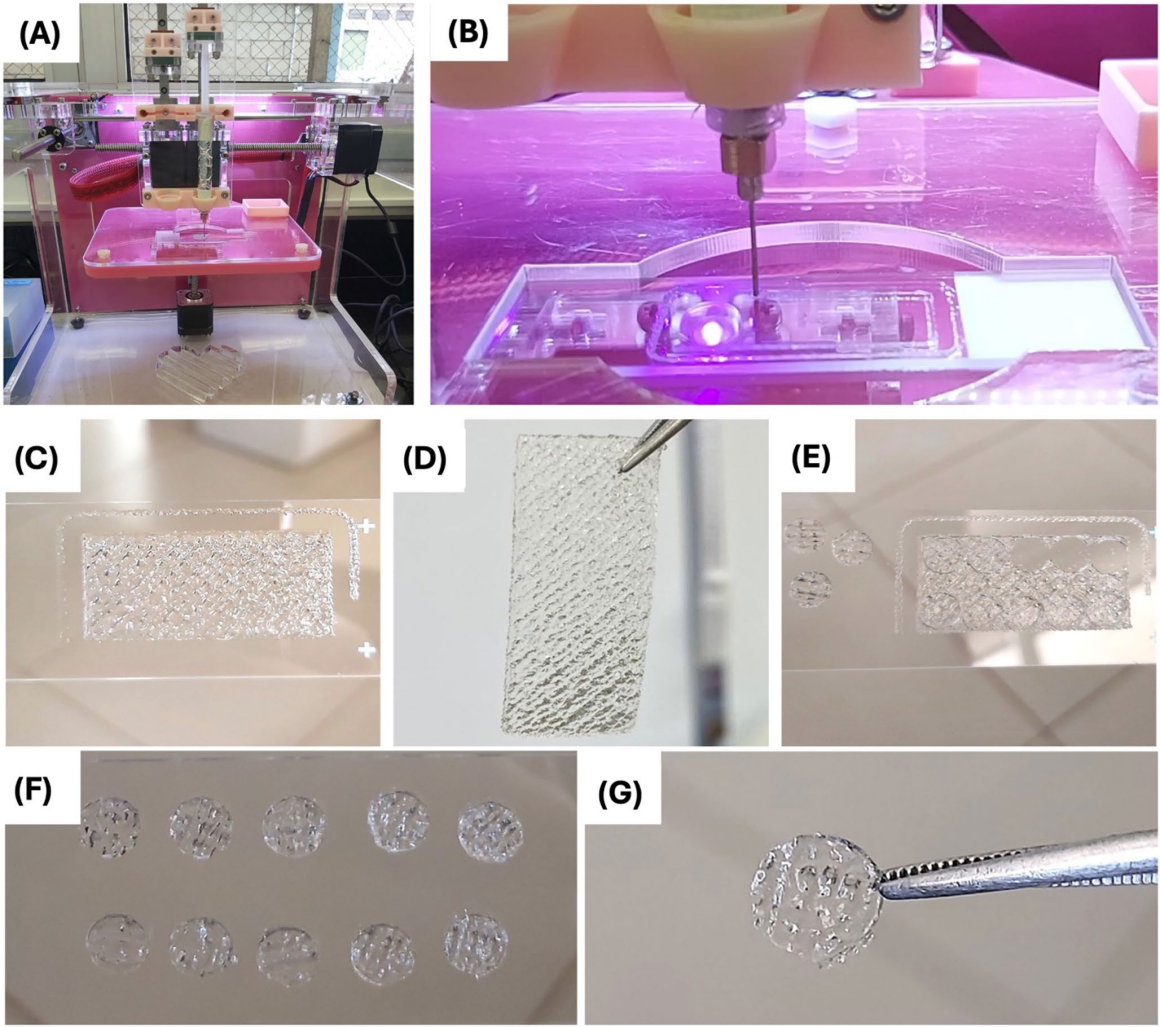
Representative images of the process of extrusion printing. (**A**) Extrusion printer used to obtain the samples; (**B**) Printing process onto a glass slide; (**C**) Rectangular shape with 50% infill of 30 mm X 15 mm X 1 mm printed on the glass slide; (**D**) Printed rectangular mesh suspended by tweezers; (**E**) Cylindrical samples cut from the rectangular mesh; (**F**) 10 cylindrical samples obtained from 1 rectangular impression; (**G**) Cylindrical sample with 6 mm in diameter

The 3D-printed hydrogel scaffold was obtained using the extrusion method with the TissueStart™ 3D Bioprinter (TissueLabs Ltda, Manno, Switzerland) (Fig. 1a and b). The extrusion-based 3D printing process was standardized to ensure reproducible filament deposition. Printing was performed at a controlled temperature of 25 °C using a 22G nozzle with a 25-mm needle length. The bioink was extruded under a constant pressure of 101.8 kPa, with a printing speed of 6 mm/s and an extrusion flow rate set to 200% relative to the baseline extrusion settings. Based on these parameters, the calculated shear rate inside the needle was approximately 8 × 10³ s⁻¹, consistent with typical shear conditions for hydrogel-based bioinks.

Each printed layer was photoactivated with LED light from the bioprinter at 405 nm. The pore diameter of the hydrogel scaffold were modulated by adjusting the internal filling of the 3D structure (infill), as determined using the Repetier-Host software (Hot-World GmbH & Co. KG, Willich, Germany). The evaluated parameters were 40%, 50%, and 60% infill. A rectangular shape of 30 mm × 15 mm × 1 mm was printed (Figs. 1c and d). Additional post-printing photoactivation was performed for 30 s using a light-emitting diode (LED) curing unit at a wavelength of 385–515 nm (1,200 mW/cm²; Bluephase N - Ivoclar Vivadent), covering 10 regions and distributing the light across the rectangular mesh with a distance of 10 mm. Finally, 10 cylindrical samples were obtained from each mesh using a 6 mm-diameter dermatological punch (Fig. 1e-f).

### Porosity of printed hydrogels

The hydrogels were immersed in a 0.5% (w/v) Rhodamine B solution (Sigma-Aldrich) in deionized water for 5 min. Afterward, the hydrogels were analyzed using an inverted microscope (Leica DM IRBE - INV-100). Ten images of each sample (*n* = 5) were captured at 16× magnification and analyzed with ImageJ software (National Institutes of Health, Bethesda, MD, USA) to determine the pore diameter.

### Degradation assay

Hydrogel samples (*n* = 8) were placed in 48-well plates with 1 mL of PBS, with or without type I collagenase (1 U/mL), and incubated at 37 °C for 24 h to allow maximum swelling. The initial wet weight (Wi) was then recorded after gently removing surface moisture. Samples were kept in PBS at 37 °C, and at predetermined time points (1, 3, 7, 14, and 21 days), the wet weight (Ww) was measured using the same procedure. PBS was refreshed weekly. Degradability (DG) was calculated as: DG (%) = (Ww – Wi)/Wi × 100.

### Compressive modulus test

Mechanical compression was performed to assess the strength and compressive modulus of the hydrogels. Samples from each group (*n* = 8) were tested using a universal mechanical testing machine (DL Digital Line, EMIC, Brazil) equipped with a 200 N load cell. Tests were carried out at room temperature at a compression rate of 1000 mm/min until fracture. Stress values (MPa) were calculated by dividing the maximum load by the specimen’s cross-sectional area. The compressive modulus was obtained from the initial linear region of the stress–strain curve, where stress was defined as σ = F/A and strain as ε = ΔL/L₀.

### Biological analysis

For the biological analysis, the hydrogels, printed under aseptic conditions, were placed in wells of 96-well plates. One drop of α-MEM culture medium (Minimum Essential Medium Eagle Alpha; Gibco, Invitrogen) containing 1 × 10⁴ human dental pulp cells (HDPCs; CAEE: 53489421.9.0000.5417. The participant donating the tooth for cell culture gave Informed Consent) was added onto the surface of the scaffolds, and the HDPC/scaffold constructs were cultivated in α-MEM culture medium (Gibco, Invitrogen), supplemented with fetal bovine serum (FBS; Gibco, Invitrogen), 1% L-glutamine, and 1% penicillin-streptomycin (Gibco, Invitrogen). The cells were then incubated at 37 °C in a 5% CO₂ environment. As a negative control (representing 100% of cell parameters), plain GelMA hydrogel (100 µL) was injected into 96-well plates and photoactivated for 30 s using LED light (385–515 nm, 1,200 mW/cm²; Bluephase N - Ivoclar Vivadent), as previously described by Silva et al. (2024) [24].

#### Cell viability and proliferation over time

After 1, 3, 7, 14, and 21 days (*n* = 2), the samples were washed in PBS (Gibco, Invitrogen) and incubated with α-MEM culture medium (Gibco, Invitrogen) supplemented with the Live/Dead cell viability/cytotoxicity kit (Invitrogen, San Francisco, CA, USA). Live (Calcein AM-positive) and dead (Ethidium homodimer-1-positive) cells on the hydrogels were observed under a fluorescence microscope (FLoid^®^, Life Technologies, Carlsbad, CA, USA) and ten images of each sample were obtained. To quantify metabolically viable cells, the Alamar Blue assay was performed after 1, 3, 7, 14, and 21 days of cultivation (*n* = 6). In each analysis period, the hydrogels were incubated for 3 h at 37 °C and 5% CO₂ in an α-MEM (without FBS) + 10% Alamar Blue^®^ reagent (Invitrogen) solution. Afterward, the supernatant was transferred to 96-well plates, and fluorescence was measured at 540 nm excitation and 590 nm emission (Synergy H1, Biotek, Winooski, USA). After this assay, GelMA and GelMA-BMdc groups with 50% and 60% infill were further evaluated, with injected GelMA hydrogel (non-printed) serving as control.

#### Cell adhesion and spreading (F-actin)

 After 1, 3, 7 and 14 and 21 days of cultivation (*n* = 2), the HDPCs/scaffold constructs were rinsed with PBS (Gibco, Invitrogen), fixed in 4% paraformaldehyde (PFA; Sigma-Aldrich), and then exposed to the fluorescent probe Alexa Fluor Phalloidin 555 (1:50; Life Technologies) for 20 min. Subsequently, nuclear counterstaining was performed using DAPI (ProLong, Thermo Fisher Scientific, Waltham, MA, USA), and five images of each sample were obtained using confocal microscopy at 20x magnification (Leica TCS SPE, Confocal Microscope).

#### Alkaline phosphatase (ALP) activity

The samples seeded with HDPCs (*n*= 6) in osteogenic medium (α-MEM supplemented with FBS, 50 µg/mL ascorbic acid, and 10 mmol/L β-glycerophosphate) were incubated and analyzed using the Alkaline Phosphatase (ALP) Activity Final Point Assay kit (Labtest Diagnostic S.A.) after 14 days of cultivation. First, the samples were lysed in a 0.1% sodium dodecyl sulfate (SDS) solution (Sigma-Aldrich) under piston maceration, followed by centrifugation at 4,000 rpm for 10 min. Next, the supernatant was collected and transferred to tubes containing the substrate thymolphthalein monophosphate (22 mmol/L, pH 10.1; Labtest Diagnostica S.A.; Lagoa Santa, MG, Brazil). The color reagent (94 mmol/L sodium carbonate and 250 mmol/L sodium hydroxide; Labtest Diagnostica S.A.; Lagoa Santa, MG, Brazil) was added, and absorbance was measured at 590 nm (Synergy MX, Biotek). The ALP activity value was obtained by dividing the ALP dosage value by the total protein value, which was quantified by adding Folin’s Solution and Ciocalteau’s Phenol Reagent (Sigma-Aldrich) for 30 minutes. Absorbance was read at a wavelength of 655 nm[30].

#### Evaluation of mineral deposition (Alizarin Red)

The Alizarin Red test was performed after 21 days of cultivation of the HDPCs-hydrogel constructs in osteogenic medium. To evaluate mineral deposition by the HDPCs, the samples (*n* = 6) were fixed in 70% ethanol at 4 °C for one hour, washed with deionized water, and incubated with an Alizarin Red solution (40 mM, pH 4.2; Sigma-Aldrich) under agitation for 15 min. Following this, the hydrogels were washed five times with deionized water, and digital photographs were captured. Next, a solution of cetylpyridinium chloride (10 mM, pH 7.0; Sigma-Aldrich) was applied for 15 min to dissolve the mineral nodules, and the absorbance of the resulting solution was measured at 560 nm (Synergy MX, Biotek). Hydrogel samples without cells served as the background control (*n* = 6).

### Artificial pulp chamber assay

**Fig. 2:**
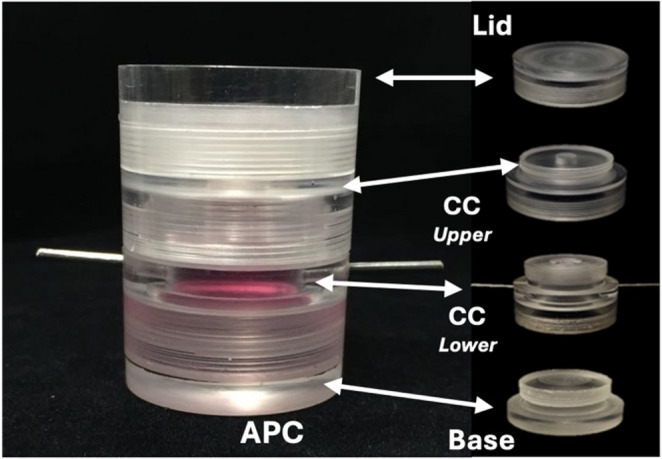
Representative images of Artificial Pulp Chamber (APC) and its parts

**Fig. 3:**
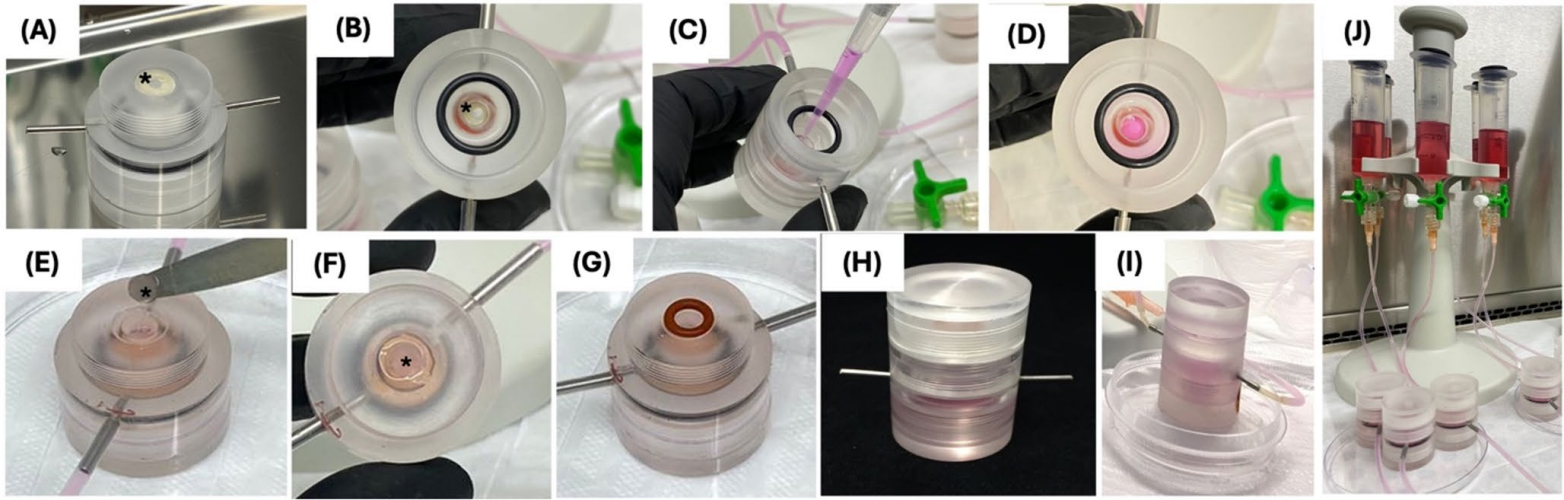
Representative images of APC experimental design. (**A**) Upper view of lower CC with dentin disc (*) containing a central perforation; (**B**) Dentin disc pulpal surface (*) view from inside lower CC chamber; (**C**) Collagen solution containing HDPCs application inside lower CC chamber; (**D**) 3D culture view after gelification; (**E**) GelMA (*) being placed onto dentin disc; (**F**) Upper view of GelMA (*) adapted onto dentin; (**G**) Silicon ring placed to promote adequate sealing; (**H**) APC view after thearing all parts and with CC filled with culture medium; (**I**) APC with lateral connections to pressure system; (**J**) Pressurized system

To evaluate the cell-hydrogel interaction in a cell-homing strategy within a biomimetic model, the artificial pulp chamber (APC) developed by our group and previously described by Soares et al. (2021) [31] was used. Its mechanism simulates the normal fluid pressure of human pulp tissue. The APC consists of a lid, a central chamber (CC), and a base (Fig. 2). A human dentin disc containing a 1 mm diameter central perforation is placed between upper and lower CC compartments (Fig. 3A and B). A HDPC 3D cell culture [31] is placed inside the lower compartment of the CC in direct contact with pulpal side of dentin disc (Fig. 3C and D). The 3D culture was established by dissolving a 3.7 mg/mL type 1 collagen solution (Corning Inc., Somerville, MA, USA) in a 4:1 ratio with a 10× concentrated α-MEM culture medium (Gibco, Invitrogen). To achieve a pH of 7.2, 5 M sodium hydroxide (Sigma-Aldrich) was used for neutralization in a 4 °C environment [31]. Next, 1 × 10⁶ HDPCs in 5 µL of medium were added to 200 µL of the collagen solution. The resulting mixture was applied to the central chamber (CC) of the APC (Fig. 3C) and incubated at 37 °C and 5% CO₂ for 30 min to allow the collagen matrix to solidify into a gel (Fig. 3D). The 3D culture was incubated without pressure for 48 h to ensure stable culture conditions. After this period, the selected hydrogels were placed into the dentin perforation (Fig. 3E and F), stablishing direct contact with the 3D culture, and then covered with polystirene. A silicon ring is placed onto the polystyrene (Fig. 3G) and all parts of APC are threaded (Fig. 3H). To stablish simulated pressure the lateral connections are coupled to the pressure system (Fig. 3I), that includes a syringe with culture medium to create the liquid column (15 cm of liquid), coupled to the APC inlet connection, providing hydrostatic pressure of 20 cm/H2O (i.e., 14.7 mm Hg), which represents the natural pulp (Fig. 3J) [32, 33]. The pressurized system was incubated at 37 °C and 5% CO₂, with daily perfusion of 5 mL culture medium to renew the medium in the system for up to 14 days. For this assay, the following groups were stablished: GelMA – injected GelMA; GelMA-BMdc – injected GelMA-BMdc; GelMA 60% − 3D printed GelMA with 60% infill; GelMA-BMdc 60% − 3D printed GelMA-BMdc with 60% infill. For all the APC analysis, *n* = 4 was used, reflecting the methodological complexity of this model, in which each chamber functions as an independent biological replicate.

#### Cell viability and proliferation

After 1, 3, 7, and 14 days (*n* = 4), the Live/Dead assay was performed as described previously, and five images of each scaffold and 3D culture were captured using a fluorescence microscope (FLoid^®^, Life Technologies). Cell metabolism of 3D culture was monitored at 3, 7, and 14 days (*n* = 4) using Alamar Blue Assay.

#### Cell migration to scaffold

The migrating cells on the surface of the hydrogels were counted after 14 days of cultivation. Ten images per sample (*n* = 4) were obtained using a fluorescence microscope (FLoid^®^, Life Technologies) after incubation with the Hoechst fluorescent probe (1:10,000; Life Technologies). The number of migrating cells was quantified using ImageJ software (National Institutes of Health).

### Statistical analysis

After assessing normality and homoscedasticity using the Shapiro–Wilk test, data were analyzed using Prism 8 software (GraphPad; San Diego, CA, USA). One-way ANOVA, followed by the Tukey post hoc test, was used to identify significant differences between groups. A p-value of < 0.05 was considered statistically significant.

## Results

### Porous architecture

**Fig. 4:**
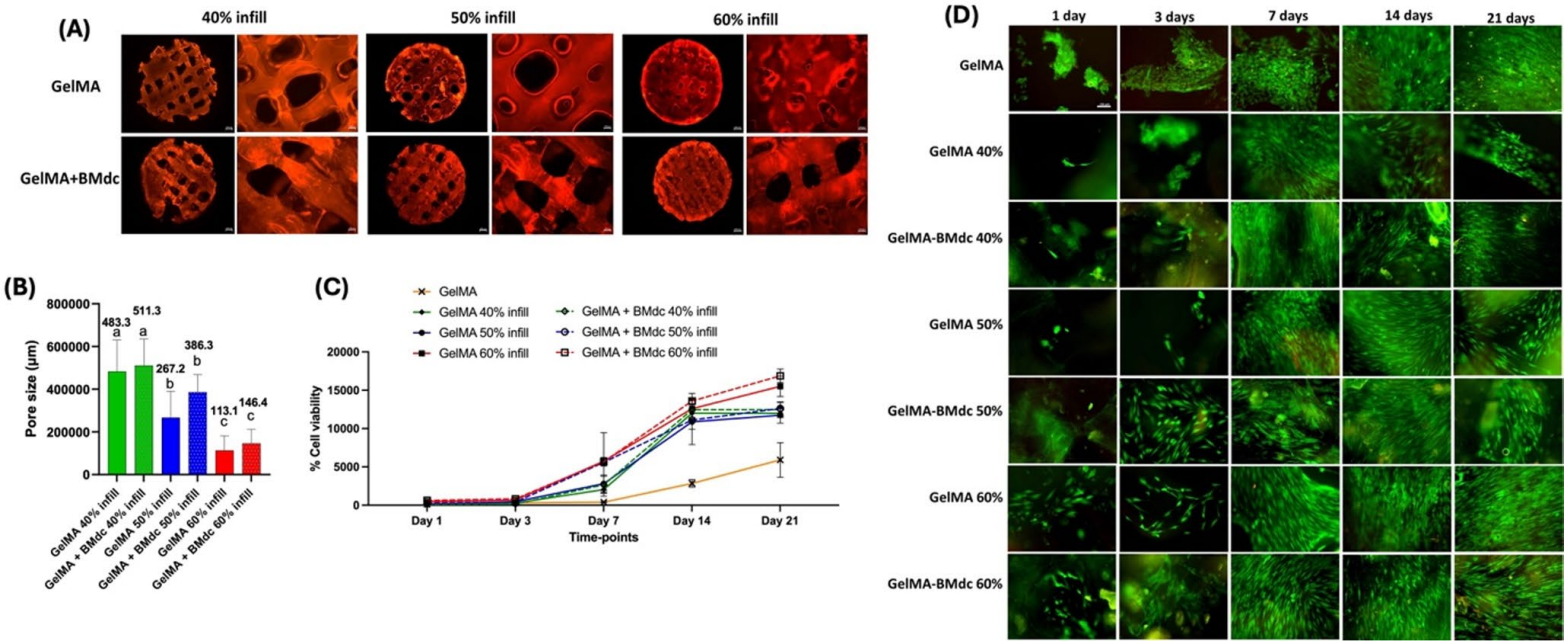
Scaffold porosity and biological characterization. (**A**) Rodamin B-stained 3D printed scaffolds according to hydrogel composition and infill %, at 2.6x and 5x magnification; (**B**) Bar graph of mean values of pore size from 3D printed scaffolds. Numbers are mean values. Different letters allow for statistic comparisons (one-way ANOVA; Tukey’s test. *P* < 0.05). (**C**) Alamar Blue assay. Representative graph of the evolution of cell proliferation for 21 days. (**D**) Live/Dead assay. Representative images of the surface of hydrogels (20x). Green: Viable cells; red: dead cells

All the 3D-printed hydrogels exhibited a macroporous structure, as observed in the Rhodamine B-stained samples (Fig. 4A). The pore size was proportional to the infill percentage, as anticipated. However, at 40% infill, the samples were more fragile due to the larger printed pores, resulting in a more disorganized printed pattern (Fig. 4B).

### Biological characterization

**Fig. 5:**
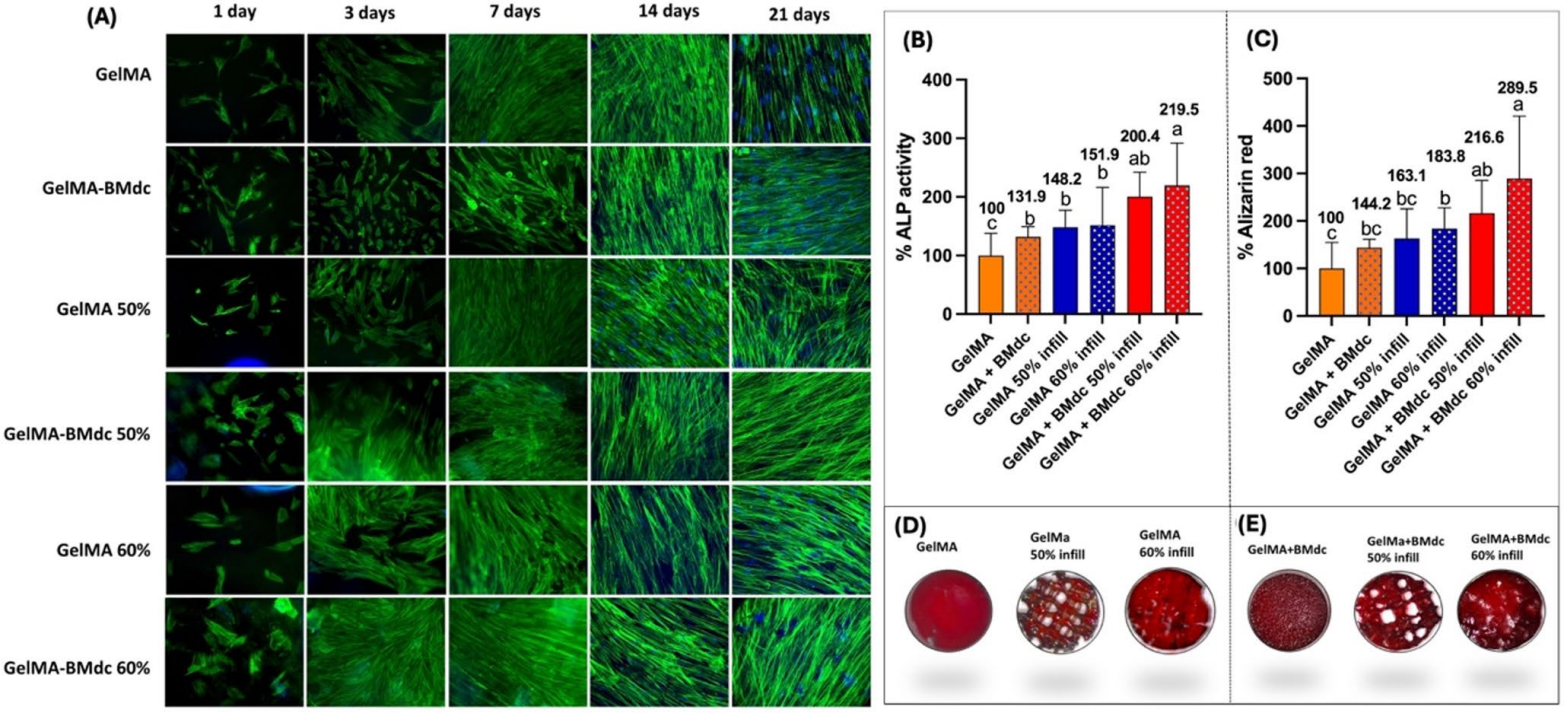
Biological characterization - Cell spread and differentiation. (**A**) Representative images of the cell adhesion and spreading assay (F-Actin; 20x). Green: actin filaments; Blue: cell nuclei; (**B**) Representative bar graph of percentage of ALP activity at 14 days. Bars represent mean and standard deviation (*n* = 6); (**C**) Bar graph representing mineral deposition marked by the Alizarin Red assay at 21 days. Bars represent mean and standard deviation (*n* = 6). Values represent average and different letters indicate a statistical difference between groups (One-way ANOVA; Tukey´s test. *p* < 0.05); (**D**) Images of samples of plain GelMA groups stained by Alizarin Red and (**E**) Images of GelMA-BMdc groups stained by Alizarin Red

As shown in Fig. 5C and D, all groups supported cell viability and proliferation. Notably, among the printed groups, the 40% infill demonstrated a lower number of viable cells compared to the 50% and 60% infill groups, particularly at the 14- and 21-day time points, as observed in the Live/Dead images (Fig. 4D). Consequently, the 40% infill group was excluded from subsequent experiments.

The F-actin assay allowed for the observation of HDPCs actin filaments (Fig. 5A), demonstrating cell adhesion and spreading on all hydrogels throughout the 21-day analysis period. However, a greater quantity of filaments was observed in the GelMA-BMdc 60% infill group at the 3- and 7-day time points. The differentiation rate was significantly influenced by the modulation of porosity through 3D printing and the addition of the bone matrix, in comparison to plain GelMA (Figs. 5B-E). The 3D-printed samples exhibited higher alkaline phosphatase (ALP) activity and greater mineralized matrix deposition compared to the injected GelMA, with the 60% infill group showing statistical difference (*p* < 0.05). The incorporation of BMdc also increased the expression of odontogenic markers compared to the injected BMdc samples, with significant difference only detected in the 60% infill group (*p* < 0.05). Digital images of Alizarin Red-stained samples at 21 days (Figs. 5D-E) indicate that the 50% infill samples appeared more degraded, while the 60% infill remained stable over time.

### Physical characterization of selected samples

**Fig. 6:**
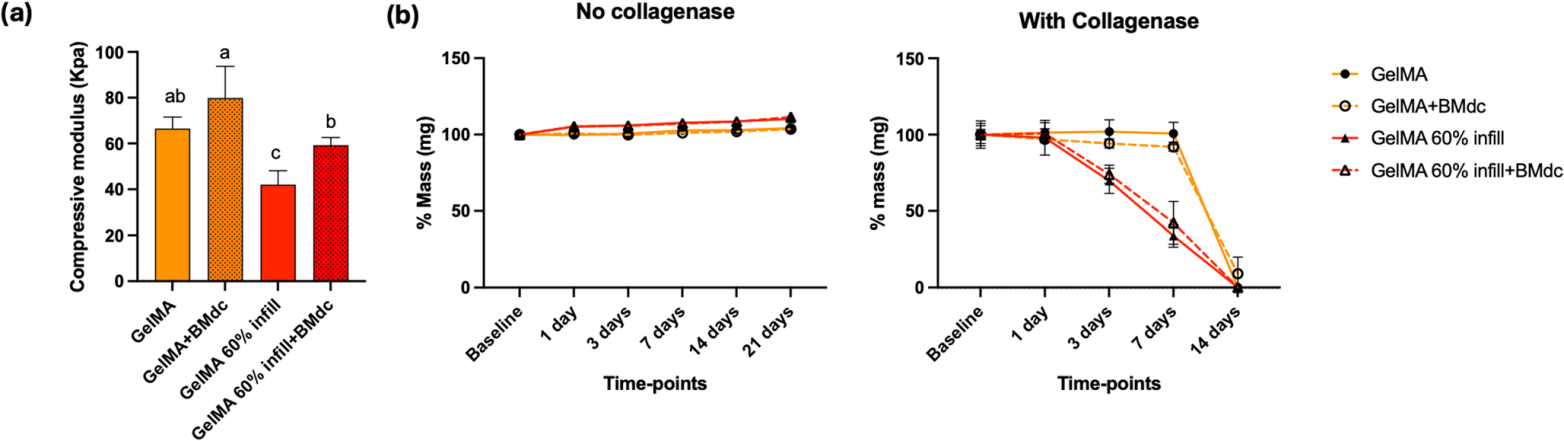
Physical characterization. (**a**) Bar graph representing compressive modulus. Bars represent mean and standard deviation (*n* = 6). Different letters indicate a statistical difference between groups (One-way ANOVA; Tukey´s test. *p* < 0.05); (**b**) Representative graph of the humid mass (%) of scaffolds in absence or presence of collagenase

The compressive modulus of GelMA and GelMA + BMdc was significantly higher than that of GelMA 60% infill. However, GelMA 60% infill + BMdc showed a significantly higher modulus than GelMA 60% infill and was statistically comparable to GelMA (Fig. 6a). No degradation was observed in any group in the absence of collagenase. Under enzymatic conditions, printed samples initiated degradation at day 1, whereas injected samples began degrading at day 7. All samples were fully degraded by day 14 (Fig. 6b).

### APC assay

**Fig. 7:**
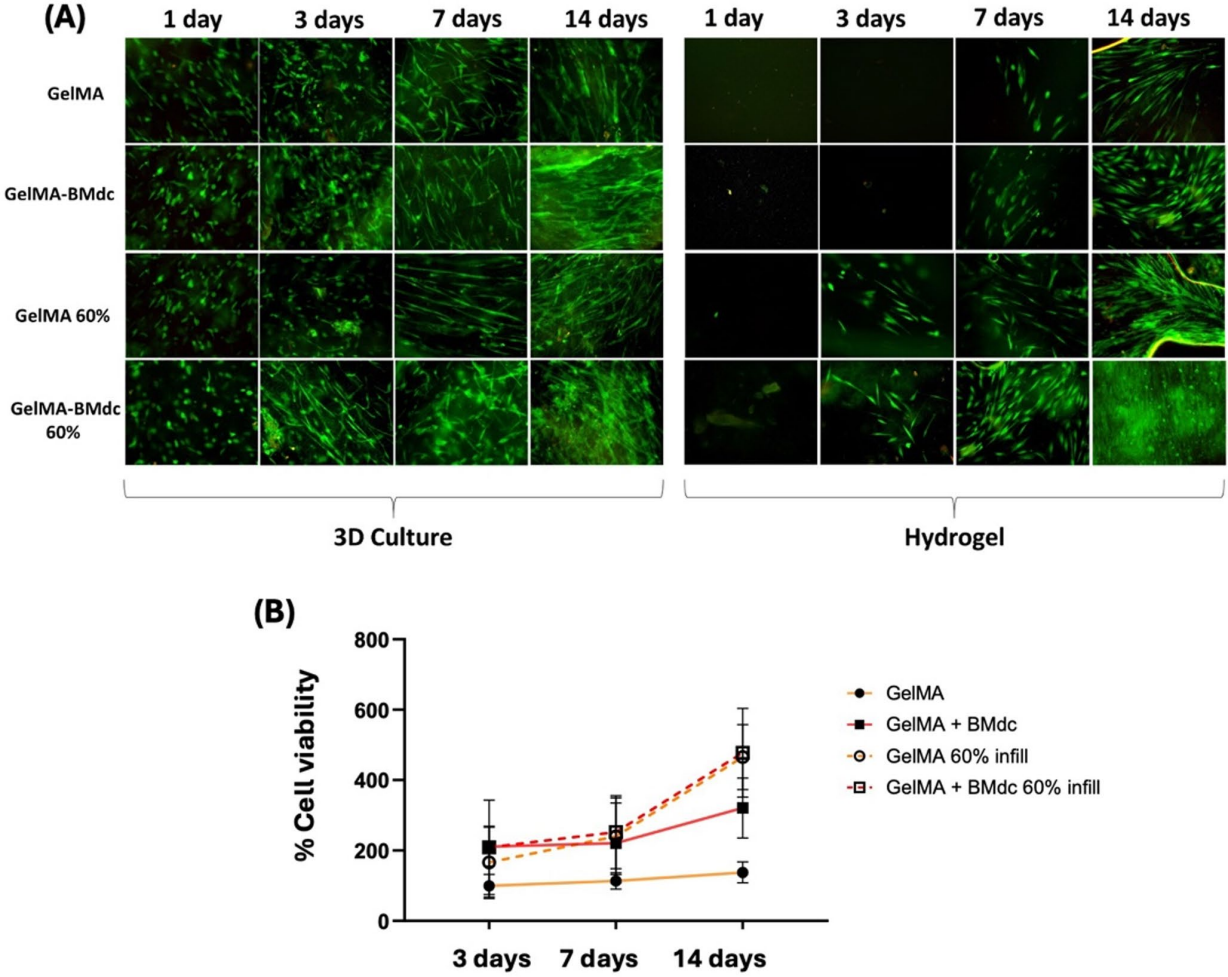
Cell viability and proliferation assay. (**A**) images of Live/Dead assay performed on hydrogels and 3D cultures (20x) grown in pAPC for up to 14 days. Green: living cells; Red: dead cells; (**B**) Representative graph of the percentage of cellular metabolism (Alamar Blue)

**Fig. 8:**
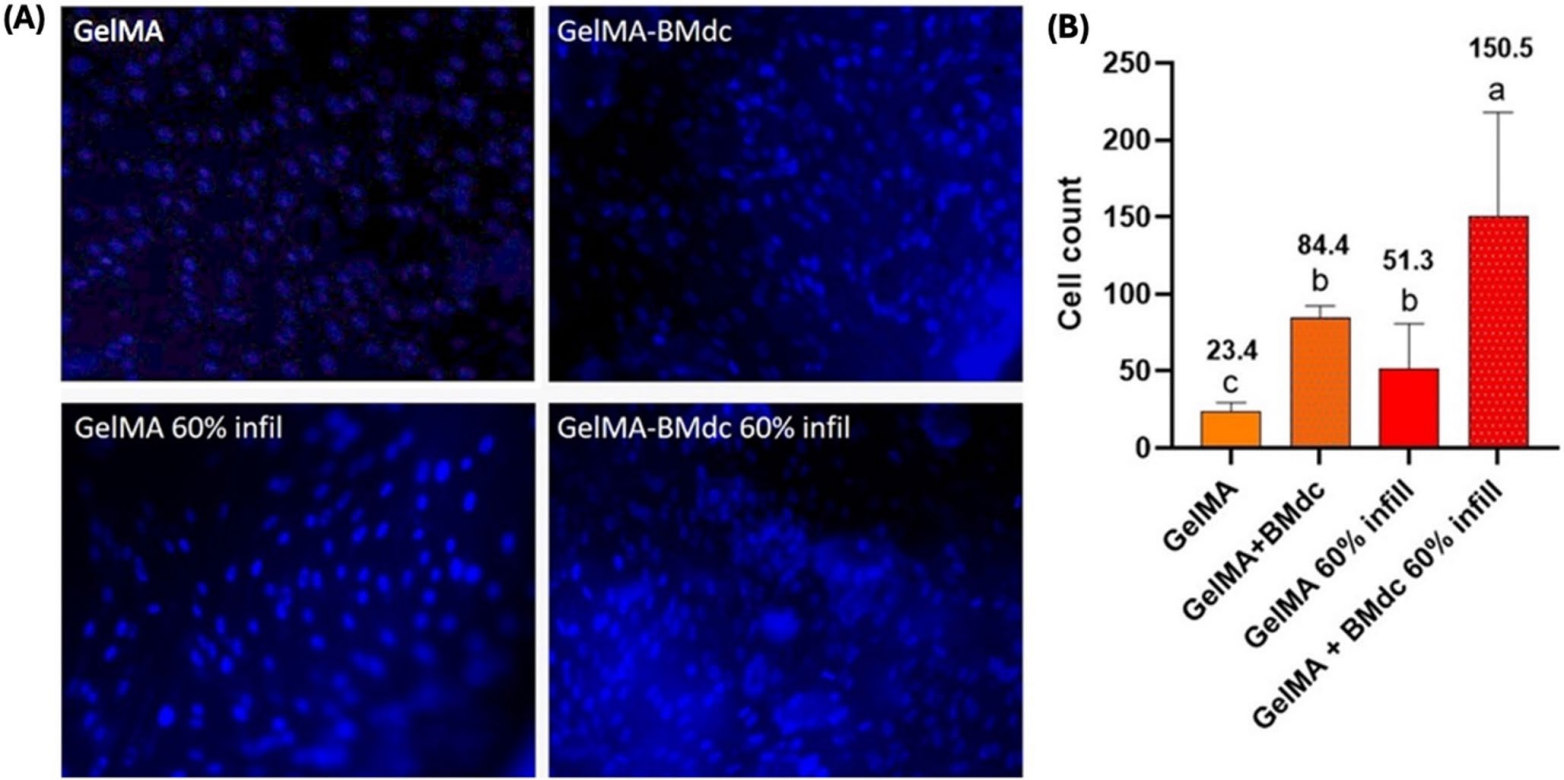
Cell migration assay. (**A**) Images obtained by fluorescence microscopy representing cells migrating to the surface of the hydrogels (20x). (**B**) Representative graph of the number of cells migrating to the surface of the hydrogels (One-way ANOVA; Tukey´s test. *p* < 0.05)

For this assay, the 60% infill pattern was selected based on previous analysis. Living cells were observed in the 3D culture of all groups throughout the 14-day period (Fig. 7A). Regarding the hydrogels, viable cells were detectable in the non-printed control groups only after 7 days of analysis, whereas in the printed groups, live cells were already observed 3 days after being seeded. At 14 days, the GelMA-BMdc 60% group exhibited a notably higher number of live cells on the hydrogel surface. The presence of BMdc and the application of 3D printing increased the number of metabolically active cells and promoted greater cell migration compared to the control group (plain GelMA), as observed in the Fig. 7B. Finally, Fig. 8 shows the quantification of migrating cells from the 3D culture to the hydrogel surface. Cell migration was significantly influenced by both 3D printing and the incorporation of BMdc. The GelMA-BMdc 60% infill group demonstrated a significantly greater ability to stimulate cell migration compared to the other groups (*p* < 0.05).

## Discussion

GelMA hydrogel has demonstrated suitability as a bioink for 3D printing applications [34, 35]. In this study, a decellularized bone matrix (BMdc) was incorporated as a signaling factor within the GelMA hydrogel to develop a bioactive bioink for 3D printing, specifically aimed at dentin tissue engineering. The composition of the bioink and the BMdc particles used in this study were previously characterized by Silva et al. (2024) [24]. BMdc particles were obtained using a protocol adapted from Grayson et al. (2008),[36] containing both organic and inorganic components, with a 99.6% efficiency in DNA removal. Silva et al. (2024) [24] also demonstrated the absence of cells in the BMdc particles following decellularization, as confirmed by fluorescence microscopy. In the present investigation, we further confirmed that this protocol effectively eliminated cellular components while preserving the organic content (Supplementary Material). Nevertheless, the translation of a novel bovine-derived scaffold, such as GelMA–BMdc, would require comprehensive assessments of immunogenicity, endotoxin levels, and batch-to-batch consistency.

Silva et al. (2024) demonstrated that the incorporation of BMdc particles enhanced the bioactive potential of GelMA, surpassing that of bovine bone ceramic obtained through conventional physicochemical processing, which ensures the complete removal of organic matter. At a concentration of 1% (w/v), the addition of BMdc to 15% GelMA supported the viability and proliferation of human dental pulp cells (HDPCs) within the hydrogel matrix, with these cells exhibiting increased expression of odontoblastic markers. Importantly, the injectability, porosity, pore size, degradability, and density of the hydrogel were not adversely affected by BMdc incorporation. The BMdc particles were uniformly integrated within the hydrogel while preserving its porous architecture, an essential feature for cell migration, nutrient diffusion, and extracellular matrix deposition. The incorporation of BMdc also resulted in an increased compressive modulus, indicating enhanced mechanical resistance of the biomaterial. In contrast, the incorporation of 1% nHA into GelMA led to obstructed pore openings and reduced pore size. Furthermore, FTIR analyses revealed chemical interactions between BMdc and GelMA amide groups, suggesting a more cohesive material interface compared with the predominantly physical incorporation observed for nHA. Based on these results, GelMA hydrogel incorporated with 1% BMdc was selected and evaluated in the present study as a bioink for dentin tissue engineering. Nevertheless, the absence of a bioceramic control can be considered a limitation of this study.

Firstly, GelMA and GelMA-BMdc hydrogels were 3D printed with three different internal filling densities—40%, 50%, and 60%—to modulate pore size and porosity. The viability and proliferation of cells in contact with the scaffolds were assessed by Live/Dead and Alamar Blue assays. Cytocompatibility was observed in all experimental groups, in which adhesion, spreading, and proliferation of HDPCs occurred over a 21-day period. Notably, the 60% infill groups exhibited a higher cells proliferation rate at day 21. On the other hand, decreased cell viability was noted in the 40% infill groups at days 14 and 21, that was attributed to the faster degradation of the biomaterials due to its lower infill density. Oversized pores and thinner walls observed in the 40% infill scaffolds, as determined by the Rhodamine B assay, may compromise the mechanical integrity of the hydrogel and lead to structural collapse[37, 38], thereby interfering with the support necessary for continued cell growth. This hypothesis warrants further investigations through degradation analysis. Consequently, the 40% infill group was excluded from subsequent experiments.

The incorporation of BMdc into the GelMA hydrogel effectively stimulated odontogenic differentiation and mineral deposition by HDPCs, particularly at the 60% infill concentration, as evidenced their increased alkaline phosphatase (ALP) activity. These effects may be attributed to the synergistic interaction of BMdc incorporated to the biomaterial and its optimized porosity. Additionally, the improved mechanical resistance demonstrated by Silva et al. (2024) [24] may contribute to an increase in scaffold stiffness, thereby making it a suitable platform for mineralized tissue regeneration, as previously suggested by Bottino et al. (2017) [39]. Similar results are consistent with the findings of Yang et al. (2023),[28] who developed bioprinted scaffolds combining GelMA with a porcine dental follicle-derived decellularized extracellular matrix (dECM), loaded with dental follicle cells (GelMA/dECM). The authors demonstrated that such scaffolds exhibited excellent mechanical properties, printability, biocompatibility, and potential to induce fibrogenesis and osteogenic differentiation in vivo. Similarly, Cunha et al. (2023) [29] reported that a 3D-printed GelMA microgel, supplemented with dentin matrix molecules, successfully induced odontoblastic differentiation and mineral deposition. This can be explained by the fact that ECM mediates signaling to resident cells, regulating their proliferation, migration, and differentiation. Decellularized ECM (dECM) biomaterials have the potential to stimulate tissue regeneration by providing a native-like environment. During decellularization, immunogenic cells and molecules are largely removed, while functional components such as glycosaminoglycans, glycoproteins, and cytokines are preserved, promoting cellular differentiation and, consequently, tissue regeneration [40]. 

In the present study, the pore size and porosity of the 3D-printed hydrogels were evaluated after incubation the biomaterials with a Rhodamine B fluorescent marker. It was observed that the 3D-printed hydrogels containing GelMA with 50% infill and GelMA-BMdc with 50% infill exhibited mean pore diameters of 592 μm and 698 μm, respectively, while the GelMA 60% infill and GelMA-BMdc 60% infill groups had average pore diameters of 361 μm and 355 μm, respectively. These pore sizes seem to be aligned with the ideal parameters for mineralized tissue regeneration, which typically range from 100 to 400 μm[3, 41–43]. Due to their higher water content, hydrogels possess a microporous cross-linking structure that not only provides structural support but also facilitates the retention of water and nutrients. In this context, the use of GelMA-BMdc 3D-printed hydrogels has proven effective in optimizing the porous structure, creating mesoscale pores larger than 100 μm, which promotes nutrient circulation, stimulates cell migration, and provides adequate space for new tissue formation, key processes for cell proliferation [30, 44–47]. In support of these findings, Soares et al. (2020) [30] demonstrated that increasing the pore diameter of chitosan scaffolds from 86.9 μm to 202.1 μm using Ca(OH)_2_ stimulated odontogenic differentiation and calcium-rich matrix deposition by dental pulp cells.

Based on the scientific data initially obtained in this laboratory study, a 60% infill parameter was selected for further analysis using a biomimetic strategy. The results demonstrated that 3D-printed scaffolds with this configuration exhibited a reduced compressive modulus compared with the injected hydrogel, which was expected, as the increased overall porosity leads to a larger surface area [28, 29]. Nevertheless, the incorporation of BMdc particles enhanced the mechanical resistance of the 3D-printed samples, reaching values comparable to those of non-printed GelMA. Degradability was not influenced by BMdc incorporation but rather by the architectural framework provided by 3D printing, which is also consistent with the increased surface area of the printed constructs [28, 29]. 

The artificial pulp chamber (APC) model used in this study effectively simulated human intrapulpal pressure, and the inclusion of type I collagen in the 3D culture seemed to turn this methodology closer to the physiologic environment of the vital pulp tissue. In a previous investigation, Soares et al. (2021) [31] demonstrated that chitosan-calcium scaffolds combined with simvastatin was capable of inducing chemotaxis to pulp cells, which showed enhanced expression of odontoblastic markers when in contact with a 3D culture adapted to the APC. These interesting results that were validated in vivo, highlight the potential of the biomimetic strategy used by the authors to replicate a natural pulp tissue environment effectively. In applying this contemporary methodology to the present study, HDPCs from the 3D culture in contact with the 3D-printed hydrogels remained viable and proliferated throughout the 14-day analysis period. All groups stimulated cells migration into the scaffolds, with the GelMA-BMdc 60% infill group showing the most noticeable results. Viable cells were observed on the surface of the printed hydrogels as early as 3 days, whereas in the non-printed hydrogels used as controls, viable cells were only detected after 7 days. This specific result suggests that modulating the porosity of 3D-printed hydrogels through 3D printing enhances early cell migration, with the GelMA-BMdc 60% infill group exhibiting superior outcomes under simulated pulp pressure. Nonetheless, because a no-pressure control was not included, it is not possible to definitively distinguish chemotaxis from pressure-driven migration.

Another limitation of this study refers to fetal bovine serum (FBS) used in cell culture experiments. FBS is widely used as a standard supplement for in vitro culture of dental pulp cells from human and animal orgin, as it effectively supports cell viability and phenotype maintenance while ensuring experimental robustness and comparability with previous studies in pulp–dentin tissue engineering[48, 49]. Nevertheless, its xenogeneic origin and batch-to-batch variability limit translational applicability of the results found in the present investigation. Additionally, although decellularized bone matrix is known to retain bioactive molecules such as growth factors, the present study did not directly quantify growth factor release from BMdc, which represents a limitation and should be addressed in future investigations. Animal-derived biomaterials, including bovine bone matrices and collagen membranes, are already used clinically with established safety profiles[50].

Despite the artificial pulp chamber providing a biomimetic environment that partially reproduces in vivo conditions, results from in vitro models cannot be directly translated to clinical scenarios. The system still lacks key biological components such as immune cells, vascularization and innervation, which play central roles in pulp–dentin regeneration. Immune cells regulate inflammation-driven odontogenic differentiation, while vascular networks support nutrient exchange and progenitor cell recruitment. Recent advances in regenerative endodontics highlight the importance of more physiologically complex platforms, including immune cell co-cultures, pre-vascularized hydrogels and microfluidic organ-on-chip systems capable of reproducing pulp microcirculation and biochemical gradients[51, 52]. Incorporating these features in future studies will enable a more comprehensive assessment of how 3D-printed GelMA–BMdc scaffolds interact with host immune, angiogenic and connective tissue responses, thereby providing a stronger foundation for establishing their regenerative potential and advancing toward in vivo and clinical validation.

## Conclusion

The 3D-printed GelMA-BMdc hydrogel with 60% infill resulted in a material with a pore size favorable for dentin regeneration. The hydrogel exhibited biocompatibility, facilitated cell adhesion and spreading, and promoted odontogenic differentiation along with mineralized matrix deposition. These findings suggest that this biomaterial holds significant potential for future applications in direct pulp capping.

## Supplementary Information

Below is the link to the electronic supplementary material.

Supplementary Material 1 (DOCX 1.94 MB)

## Data Availability

The data that support the findings of this study are available from the corresponding author upon reasonable request.
