# Supplementary material for: Decellularized bone matrix-enriched 3D-printed GelMA scaffold as a cell-homing platform: analysis using an artificial pulp chamber model
Source: Clin Oral Investig. 2026 Feb 9;30(2):74. doi: 10.1007/s00784-026-06761-7 (PMC12883528; doi:10.1007/s00784-026-06761-7)
Supplement: Supplementary file 1 — Supplementary Material 1 (DOCX 1.94 MB) [file 784_2026_6761_MOESM1_ESM.docx]

**Supplemental Material**

**Hoechst Fluorescent Staining:** Samples were fixed, washed in PBS, and incubated with Hoechst 33342 (1:1000) for 10 minutes in the dark. After a final PBS wash, the samples were mounted on slides and examined under a fluorescence microscope (EVOS F-Loid, ThermoFisher Scientific) to qualitatively assess residual nuclear material (n=4)

**DNA quantification:** A commercial DNA Quantitation Kit (Sigma-Aldrich), according to the manufacturer’s protocol (n=6). Briefly, BMdc samples were enzymatically digested to release nucleic acids, and aliquots of the resulting lysates were incubated with the kit’s fluorescent binding reagent. Fluorescence intensity was recorded using a microplate reader (Synergy H1, Biotek)., and DNA concentration (ng/mL) was calculated from a standard curve prepared with known DNA concentrations.

**Residual protein content:** BMdc samples were first solubilized, and the resulting extracts were mixed with the alkaline copper tartrate reagent, followed by the addition of the Folin–Ciocalteu phenol reagent (n=6). After incubation, absorbance was measured at 750 nm (Synergy H1, Biotek). Protein concentration was determined from a standard curve constructed with bovine serum albumin (BSA).

**Results**

Hoechst staining (Figure 1a) confirmed the absence of positively stained nuclei after decellularization. Consistently, the residual DNA content was effectively removed (Figure 1b), while the total protein content remained unchanged (Figure 1c), indicating that the decellularization protocol was successfully performed.


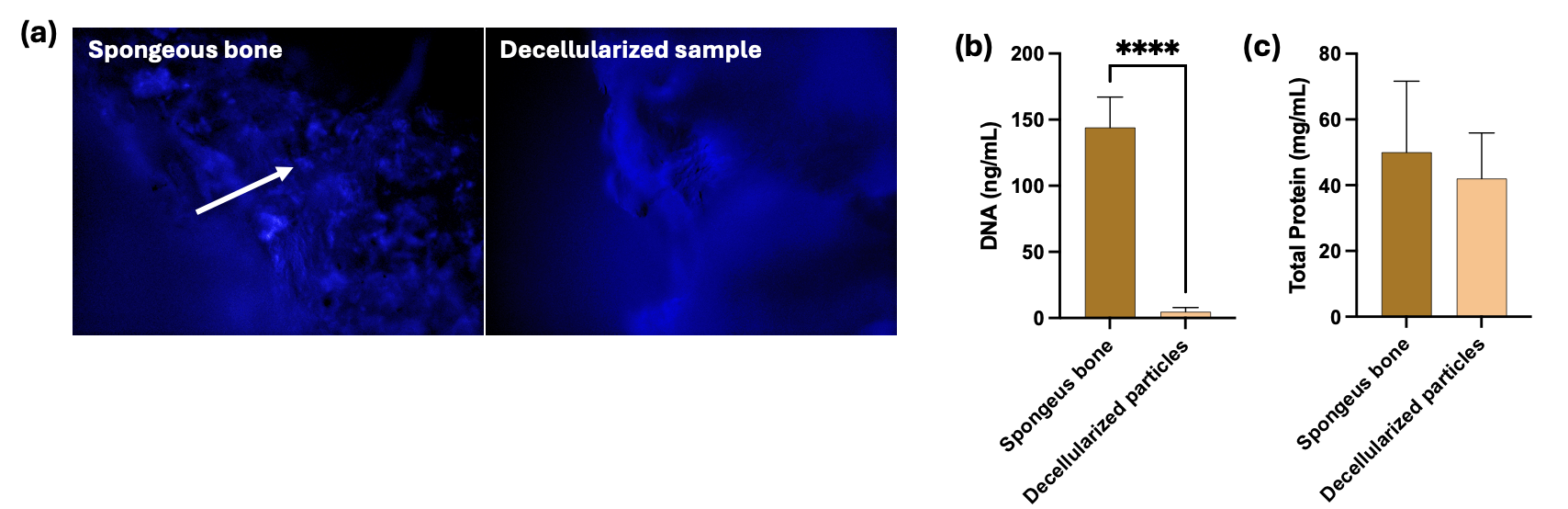


**Figure S1.** (a) Fluorescence images of samples stained with Hoechst. Arrows indicate nuclei, which are absent in decellularized samples (20×). (b) and (c) Bar graphs showing the mean (SD) DNA content and total protein levels, respectively. Statistical analysis was performed using Student’s *t*-test (P < 0.05).

**Hidrogel characterization**

**Injectability:** Injectability was assessed by extruding the hydrogels from a 3 mL syringe fitted with a 22G needle using a piston-driven bioprinter (TissueStart™ 3D Bioprinter, TissueLabs Sagl, Switzerland) operating in extrusion mode. A constant pressure of 101.8 kPa was applied, and the residual mass remaining in the syringe was recorded (n = 6). Because GelMA undergoes thermogelation, all injectability tests were standardized at a room temperature of 25 °C. Density

**Density** Hydrogel density after photoactivation was calculated using the equation d = m / v, where *d* is density, *m* is mass, and *v* is volume. For that, samples (100 uL) were injected on 96-well plates and photoactivated for 30 15 seconds using an LED light with a wavelength range of 385 to 515 nm (1,200 mW/cm2; Bluephase N, Ivoclar-Vivadent, Buffalo, NY, USA). Diameter and thickness were measured with digital paquimeter (triplicate) (n=8).

**Viscosity:** GelMA viscosity (n=8) was assessed using a Marte MVD-5 rotational viscometer designed for small-volume samples. Each formulation (5 mL) was equilibrated at **25 °C** for 10 min before analysis. The spindle was immersed to the manufacturer’s reference depth, and viscosity was recorded across a series of rotational speeds after allowing 30–60 s for signal stabilization. Apparent viscosity (mPa·s) and torque (%) were collected for each condition, and all measurements were performed in triplicate to ensure reproducibility.

**Results**

Figure 2 illustrates the extrudability of the hydrogels using the extrusion bioprinter. No significant differences were observed among the groups in terms of injectability, density, or viscosity (Table 1).


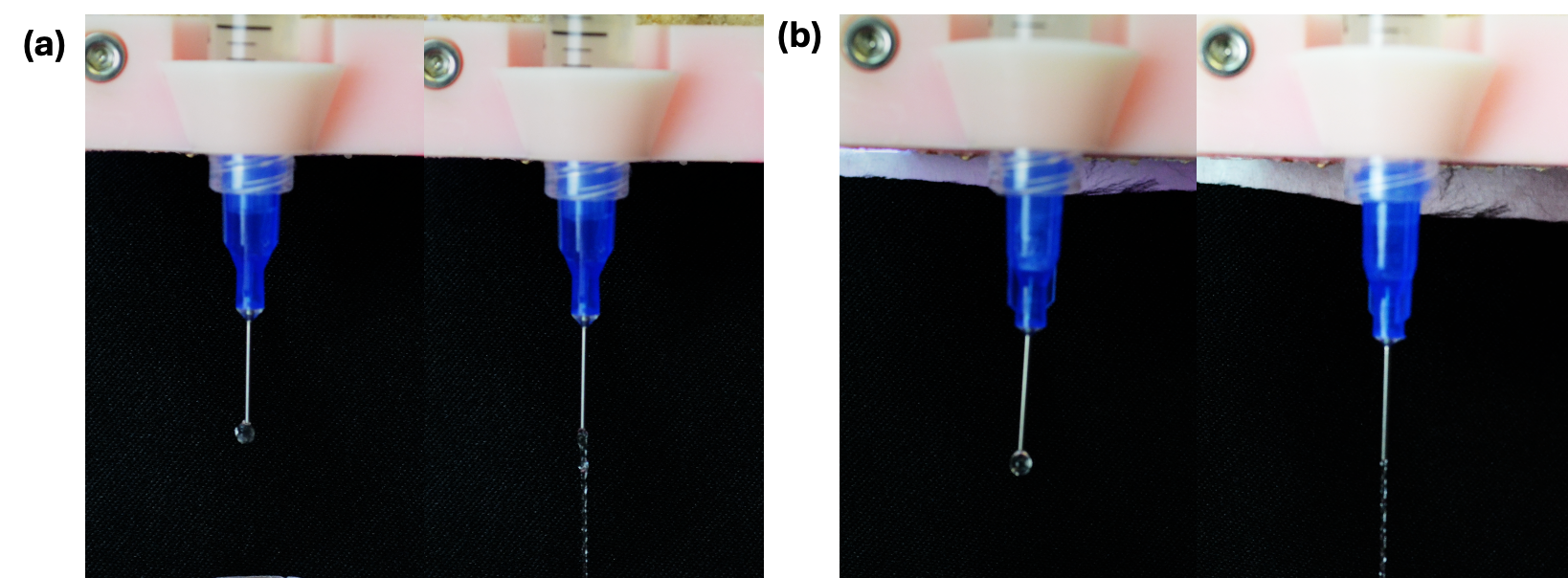


Figure S2. Representative images of hidrogels being extruded for injectability test. (a) GelMA. (b) GelMA+BMdc.

Table 1. Physical parameters of hidrogels.

| Group | Injected mass (g) | Density (g/cm3) | Viscosity (mPa.s) |
| --- | --- | --- | --- |
| GelMA | 0.270 (±0.069) | 0.648 (±0.050) | 61.53 (±8.24) |
| GelMA+BMdc | 0.269 (±0.043) | 0.655 (±0.049) | 61.69 (±8.20) |

* Statistical analysis was performed using Student’s *t*-test. No significant differences were found between groups (P > 0.05).
